# Neuroprotective Effects of Molecular Hydrogen via Oxidative Stress and Neuroinflammation Regulation in a 5xFAD Mouse Model

**DOI:** 10.3390/antiox15030404

**Published:** 2026-03-23

**Authors:** Chaodeng Mo, Johny Bajgai, Md. Habibur Rahman, Hui Ma, Thu Thao Pham, Haiyang Zhang, Buchan Cao, Eun-Sook Jeong, Cheol-Su Kim, Kyu-Jae Lee

**Affiliations:** 1 Department of Convergence Medicine, Wonju College of Medicine, Yonsei University, Wonju 26426, Republic of Koreamahui56@yonsei.ac.kr (H.M.); phamthuthaoytcc@gmail.com (T.T.P.); zhanghyzrc1017@gmail.com (H.Z.); caobuchan777@gmail.com (B.C.);; 2 Department of Global Medical Science, Graduate School, Wonju College of Medicine, Yonsei University, Wonju 26426, Republic of Korea; 3 Department of Laboratory Medicine, Wonju College of Medicine, Yonsei University, Wonju 26426, Republic of Korea

**Keywords:** molecular hydrogen (H_2_), hydrogen gas inhalation, Alzheimer’s disease, oxidative stress (OS), neuroinflammation, amyloid-beta (Aβ), neuroprotection, 5xFAD mouse model

## Abstract

Alzheimer’s disease (AD) is a progressive neurodegenerative disorder in which amyloid-beta (Aβ) accumulation, oxidative stress (OS), and chronic inflammation drive synaptic dysfunction and cognitive decline. Molecular hydrogen (H_2_) has emerged as a candidate neuroprotective gas with selective antioxidant and anti-inflammatory properties, although its efficacy in amyloid-driven pathology remains incompletely defined. In this study, 5xFAD transgenic mice harboring human amyloid precursor protein (APP) and presenilin-1 (PSEN1) mutations and age-matched C57BL/6 wild-type mice were exposed to 2% H_2_ by inhalation for 1 h/day over 4 weeks. H_2_ inhalation reduced hippocampal reactive oxygen species (ROS), increased systemic catalase activity, and enhanced hippocampal ATP levels. In serum, H_2_ decreased tumor necrosis factor-α (TNF-α) and interleukin (IL)-1β, restored IL-10, and partially normalized IL-13, shifting the peripheral environment toward a less pro-inflammatory profile. In the hippocampus, H_2_ upregulated nuclear factor erythroid 2-related factor 2 (NRF2), attenuated nuclear factor kappa B (NF-κB) activation, reduced the BAX/BCL-2 ratio, preserved neuronal nuclei (NEUN) expression, and decreased hippocampal Aβ42 burden. Collectively, these findings indicate that H_2_ inhalation confers multi-faceted neuroprotection in 5xFAD mice by restoring redox homeostasis, suppressing inflammation, improving mitochondrial function, and limiting Aβ accumulation.

## 1. Introduction

Alzheimer’s disease (AD) is a progressive neurodegenerative disorder characterized by cognitive decline and behavioral disturbances, with primary pathological features including extracellular amyloid beta (Aβ) deposition, intracellular neurofibrillary tangles composed of hyperphosphorylated tau protein, and widespread synaptic dysfunction with neuronal loss [1]. Epidemiological studies indicate that AD accounts for approximately 60–80% of dementia cases worldwide [2,3]. With accelerating population aging, dementia incidence continues to rise, posing an urgent public health challenge, and the number of individuals living with dementia is projected to reach approximately 131.5 million by 2050 [4]. Despite decades of research focused on Aβ and tau pathology, no disease-modifying therapies have demonstrated sustained clinical efficacy, reflecting the complexity and heterogeneity of AD pathogenesis [5].

Accumulating evidence indicates that mitochondrial dysfunction, oxidative stress (OS), and chronic neuroinflammation act in concert to drive neurodegeneration in AD [6,7,8]. In the AD brain, impaired mitochondrial oxidative phosphorylation and electron transport chain instability lead to excessive generation of reactive oxygen species (ROS), reduced ATP synthesis, and failure to meet neuronal metabolic demands [9]. Excess ROS damage mitochondrial DNA [10], membrane lipids [11], and proteins, disrupt mitochondrial membrane potential, and promote mitochondrial permeability transition pore opening [12], collectively lowering cellular tolerance to Aβ toxicity.

Under physiological conditions, the nuclear factor erythroid 2-related factor 2 (NRF2) pathway preserves intracellular redox balance by inducing detoxifying and antioxidant enzymes such as glutathione peroxidase (GPX), catalase, and heme oxygenase-1 (HO-1) [13]. In AD, persistent oxidative stress and Aβ-mediated repression of NRF2 signaling weaken endogenous antioxidant defenses, leaving neurons susceptible to dysfunction and apoptosis under chronic oxidative injury [14]. Damaged mitochondria release damage-associated molecular patterns (DAMPs), including mitochondrial DNA fragments and cardiolipin, which engage innate immune pathways such as toll-like receptors (TLRs), the cyclic GMP–AMP synthase (cGAS)–stimulator of interferon genes axis, and the NLR family pyrin domain-containing 3 (NLRP3) inflammasome [15]. The resulting chronic microglial activation, characterized by sustained nuclear factor kappa B (NF-κB) signaling and increased secretion of interleukin 1β (IL-1β), interleukin 6 (IL-6), and tumor necrosis factor α (TNF-α), exacerbates synaptic loss and neuronal death [16,17,18].

Currently approved pharmacotherapies for AD primarily provide symptomatic relief through modulation of neurotransmitter systems, including acetylcholinesterase inhibitors (donepezil, galantamine, rivastigmine) and the N-methyl-D-aspartate receptor antagonist memantine [19]. Recent advances in disease-modifying therapies have focused on targeting core pathological hallmarks using anti-amyloid monoclonal antibodies such as lecanemab and donanemab, which promote microglial-mediated clearance of Aβ plaques and demonstrate approximately 30% reduction in cognitive and functional decline in early-stage AD patients [20], alongside therapeutic strategies targeting tau pathology, including aggregation inhibitors and immunotherapies [21]. However, the multifactorial nature of AD pathogenesis suggests that interventions directed solely at protein aggregation are insufficient [22]. Emerging evidence highlights neuroinflammation and oxidative stress as key pathogenic drivers, with genome-wide association studies identifying immune-related susceptibility loci (APOE, TREM2, CD33) that modulate microglial function and inflammatory responses [23,24,25]. Therapeutic strategies targeting inflammatory and oxidative pathways, therefore, represent promising complementary approaches to address AD pathophysiology.

Molecular hydrogen (H_2_), a small gaseous molecule with unique physicochemical properties and a favorable safety profile, has attracted attention as a potential multi-target therapeutic agent for neurodegenerative disorders [26,27,28,29]. Unlike conventional antioxidants that indiscriminately scavenge ROS, H_2_ exhibits selective reactivity toward highly cytotoxic species, including hydroxyl radicals (·OH) and peroxynitrite (ONOO^−^), while preserving superoxide and hydrogen peroxide involved in physiological signaling [30,31]. Owing to its small size and amphipathic nature, H_2_ rapidly diffuses across biological membranes, including the blood–brain barrier, enabling efficient intracellular distribution and mitochondrial access [32]. Through these properties, H_2_ has been shown to modulate mitochondrial function and redox homeostasis. To date, more than 2000 scientific publications, including in vitro, animal, and human studies, have explored the therapeutic potential of H_2_ [33]. Numerous preclinical studies have demonstrated that H_2_ administration suppresses pathological ROS overproduction, stabilizes mitochondrial membrane potential, promotes mitophagy, and activates the NRF2/antioxidant response element (ARE) pathway, leading to enhanced expression of downstream antioxidant enzymes [30,32,34,35,36]. In addition to its antioxidant effects, H_2_ also exerts immunomodulatory activity. Specifically, attenuation of mitochondrial ROS by H_2_ can inhibit redox-sensitive inflammatory signaling pathways, including NF-κB activation and NLRP3 inflammasome assembly, thereby reducing the production of pro-inflammatory mediators [37,38,39]. Consistent with these mechanisms, studies in AD-related experimental models have shown that H_2_ administration ameliorates cognitive deficits, reduces lipid peroxidation in the brain, and decreases inflammatory cytokine expression, suggesting that H_2_ can simultaneously mitigate oxidative stress and neuroinflammation [40]. Clinical observations further support its systemic protective effects. For instance, H_2_ inhalation has been reported to attenuate radiation-related bone marrow injury, preserve peripheral white blood cells (WBCs), and alleviate radiotherapy-induced myelosuppression [41]. Similarly, our previous work demonstrated that H_2_ reduces airway inflammatory cell infiltration, highlighting its role in regulating peripheral immune responses and maintaining immune homeostasis [42].

From a pharmacokinetic perspective, H_2_ behaves as an inert and highly diffusible gas that is not enzymatically metabolized. Instead, it rapidly dissolves in body fluids, distributes through the circulation, and is ultimately eliminated via exhalation. Owing to its small molecular size and nonpolar nature, H_2_ can readily diffuse into mitochondria, where it attenuates excessive mitochondrial ROS generation and thereby contributes to the preservation of mitochondrial membrane potential and mitochondrial respiratory function. By stabilizing the electron transport chain and limiting ROS-induced mitochondrial damage, H_2_ helps maintain mitochondrial bioenergetic homeostasis [32,43]. Because mitochondrial ROS act as upstream regulators of redox-sensitive inflammatory pathways, modulation of mitochondrial redox balance by H_2_ is expected to influence downstream cytokine production [32,43,44]. Based on this mechanistic framework, TNF-α, IL-1β, and IL-6 were selected as representative pro-inflammatory cytokines associated with ROS-dependent inflammatory signaling, whereas IL-10 was included as a key anti-inflammatory mediator reflecting the counter-regulatory immune response. Consistent with this rationale, previous in vivo studies have reported reduced levels of TNF-α, IL-1β, and IL-6 following H_2_ administration, and in some cases increased IL-10, indicating that H_2_ modulates inflammatory cytokine profiles rather than acting as a non-specific suppressor [43,44,45]. Therefore, this cytokine panel was selected to reflect mitochondria-related inflammatory pathways that are responsive to H_2_. To investigate these mechanisms in vivo, we employed the 5xFAD transgenic mouse model to evaluate the effects of H_2_ inhalation on OS parameters, mitochondrial energy metabolism, Aβ burden, and inflammatory profiles. Through this approach, the present study aims to elucidate the molecular mechanisms underlying the coordinated antioxidant and anti-inflammatory actions of H_2_ in amyloid-associated neurodegeneration and to assess its feasibility as a multi-target intervention for AD, thereby providing experimental support for potential clinical translation.

## 2. Materials and Methods

### 2.1. Experimental Animals and H_2_ Gas Inhalation Protocol

All animals were maintained in accordance with the Institutional Animal Care and Use Committee guidelines of Wonju College of Medicine, Yonsei University, which approved all experimental procedures (ethical approval No: YWC-250728-1, Approval date: 4 August 2025). Male and female mice aged 2–2.5 months were used, including transgenic 5xFAD mice harboring human amyloid precursor protein (APP) and presenilin 1 (PSEN1) mutations and C57BL/6 wild-type (WT) mice (The Jackson Laboratory, Bar Harbor, ME, USA; RRID: MMRRC_034848-JAX; Stock No. 008730). Hemizygous 5xFAD mice were generated by mating transgenic 5xFAD mice with F1 C57BL/6 mice, and genotypes were confirmed by PCR using APP/PS1 primers (forward: 5′-ACC CCC ATG TCA GAG TTC CT-3′; reverse: 5′-CGG GCC TCT TCG CTA TTA C-3′).

**Figure 1:**
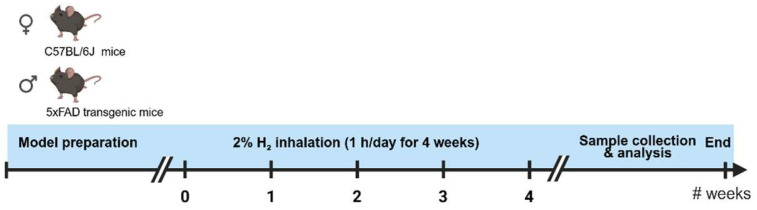
Schematic overview of the experimental design.

Mice were housed in standard cages under a 12:12 h light/dark cycle with free access to food and water. Animals were randomly allocated to four experimental groups (n = 10 per group) with balanced sex distribution (four males and six females): WT-Veh, WT-H_2_, 5xFAD-Veh, and 5xFAD-H_2_. H_2_-treated groups received 2% H_2_ by inhalation in a gas mixture containing 20.6% O_2_ and 77.4% N_2_ (totaling 98% as air), delivered using an H_2_-generating device named Hueprogen (GOOTZ Co., Ltd., Yangju-si, Republic of Korea) in a closed exposure chamber for 1 h/day over 4 consecutive weeks. Corresponding control groups underwent identical handling and environmental conditions and received normal air ventilation without H_2_ supplementation. A 2% H_2_ concentration and the 4-week inhalation protocol were selected based on previous in vivo studies demonstrating their safety and efficacy in producing antioxidant and neuroprotective effects [30,46,47]. The experimental workflow for H_2_ gas intervention is illustrated in Figure 1.

### 2.2. Tissue Collection and Sample Preparation

Following completion of the treatment period, mice were deeply anesthetized and euthanized in a CO_2_ chamber, and blood was collected from the orbital sinus. Serum was isolated by centrifugation and stored at −80 °C until analysis. Brains were rapidly removed, and hippocampi were dissected on ice, frozen in liquid nitrogen, and stored at low temperature for subsequent assays. Total protein concentrations in hippocampal lysates were determined using a bicinchoninic acid (BCA) assay kit (Thermo Fisher Scientific, Waltham, MA, USA) according to the manufacturer’s instructions. Protein concentrations were calculated from a bovine serum albumin standard curve and normalized to 2 mg/mL for subsequent analyses.

### 2.3. Analysis of Peripheral WBC Counts

Blood was collected from the retro-orbital venous plexus into ethylenediaminetetraacetic acid (EDTA)-coated BD Vacutainer tubes and mixed on an automatic roller mixer for 5 min. Total WBC counts and differential neutrophil, lymphocyte, monocyte, and eosinophil counts were obtained using a Hemavet HV950 FS automated hematology analyzer (Drew Scientific Inc., Dallas, TX, USA).

### 2.4. Assessment of OS Markers and Antioxidant Enzyme Activities

ROS levels in hippocampal tissue lysates were measured using 2′,7′ dichlorodihydrofluorescein diacetate (DCF DA; Millipore, Beijing, China), with fluorescence read at 488/525 nm using a DTX 880 microplate reader (Beckman Coulter, Brea, CA, USA). Serum nitric oxide (NO) concentrations were quantified using the Griess reagent system (Promega, Madison, WI, USA) by measuring absorbance at 540 nm. Catalase activity in serum was determined using the Catalase Activity Assay Kit (Invitrogen, Carlsbad, CA, USA), and GPX activity was measured using the Glutathione Peroxidase Assay Kit (Abcam, Cambridge, UK), following the manufacturer’s instructions for data analysis.

### 2.5. Analysis of Cytokine Profiles

Serum samples were analyzed for TNF-α, IL-1β, IL-6, granulocyte–macrophage colony-stimulating factor (GM-CSF), IL-10, and IL-13 using a commercial multiplex bead-based cytokine assay (Merck KGaA, Darmstadt, Germany). Fluorescence signals were acquired on a Luminex platform (Bio-Plex Multiplex Bead Array System™, Bio-Rad, Hercules, CA, USA), and cytokine concentrations were calculated from standard curves using the accompanying analysis software.

### 2.6. Western Blot Analysis of Hippocampal Proteins

Western blot analysis of hippocampal proteins was performed using RIPA lysis buffer (IBS BR002a, iNtRON Biotechnology, Seongnam, Republic of Korea) containing protease and phosphatase inhibitors, and total protein concentrations were determined with a BCA Protein Assay Kit. Hippocampal tissue from each of the 10 mice per experimental group was homogenized, and total protein was extracted separately. Before gel loading, equal amounts of protein from the 10 individual lysates were pooled in proportion to concentration to generate one mixed sample per group for Western blot analysis. Equal amounts of protein (10 μg per lane) were separated by SDS–PAGE and transferred to PVDF membranes. Membranes were blocked in blocking buffer (TransLab, Beijing, China) for 1 h at room temperature and incubated overnight at 4 °C with primary antibodies against NRF2 (1:10,000, Immunoway, Plano, TX, USA), HO-1, NF-κB p65 (RelA), inhibitor of nuclear factor kappa B alpha (IκBα), BCL-2 associated X protein (BAX), B cell lymphoma 2 (BCL-2), RNA binding fox-1 homolog 3 (RBFOX3; neuronal nuclei [NEUN]), and β-actin (all 1:1000, Cell Signaling Technology, Danvers, MA, USA, respective catalog numbers as listed in the Reagents section). After three washes with TBST, membranes were incubated for 1 h at room temperature with horseradish peroxidase (HRP)-conjugated anti rabbit secondary antibody (1:3000, Cell Signaling Technology). Immunoreactive bands were visualized using enhanced chemiluminescence detection reagent (Thermo Fisher Scientific, Waltham, MA, USA), and band intensities were quantified using ImageJ software (version 1.x, National Institutes of Health, Bethesda, MD, USA). For each target protein, Western blotting was repeated three times using aliquots of the same pooled lysates, and mean values from these technical replicates were used for graphical presentation and statistical analysis.

### 2.7. Quantification of Aβ40 and Aβ42 by Enzyme-Linked Immunosorbent Assay (ELISA)

Hippocampal Aβ40 and Aβ42 levels were quantified using ELISA kits (FineTest, Wuhan, China). Hippocampal tissue was homogenized in ice-cold phosphate-buffered saline (PBS) containing phenylmethylsulfonyl fluoride (PMSF), and supernatants were collected by centrifugation. Protein concentrations were measured by BCA assay and normalized to 2 mg/mL. Standards and samples were applied to pre-coated 96-well plates, processed according to the manufacturer’s protocol, and absorbance at 450 nm was measured to calculate Aβ40 and Aβ42 concentrations from standard curves.

### 2.8. Determination of Hippocampal ATP Levels

Hippocampal ATP levels were quantified using an ATP Assay Kit (Abcam, Cambridge, UK) following the manufacturer’s instructions. Hippocampal tissue was homogenized in ice-cold PBS containing PMSF, and supernatants were normalized for protein content determined by BCA assay before ATP measurement. Samples were deproteinized using a Deproteinizing Sample Preparation Kit (Abcam, Cambridge, UK) and processed in 96-well plates. Absorbance at 450 nm was measured to calculate ATP concentrations from standard curves and expressed as nmol ATP per mg protein.

### 2.9. Statistical Analysis

Statistical analyses were conducted using GraphPad Prism 10.1.2 (GraphPad Software, La Jolla, CA, USA). Data were analyzed by one-way analysis of variance (ANOVA) with Tukey’s post hoc multiple comparison test, and *p* < 0.05 was considered statistically significant. Results are presented as mean ± standard deviation (SD).

## 3. Results

### 3.1. Effects of H_2_ in Peripheral WBC Counts

**Figure 2:**
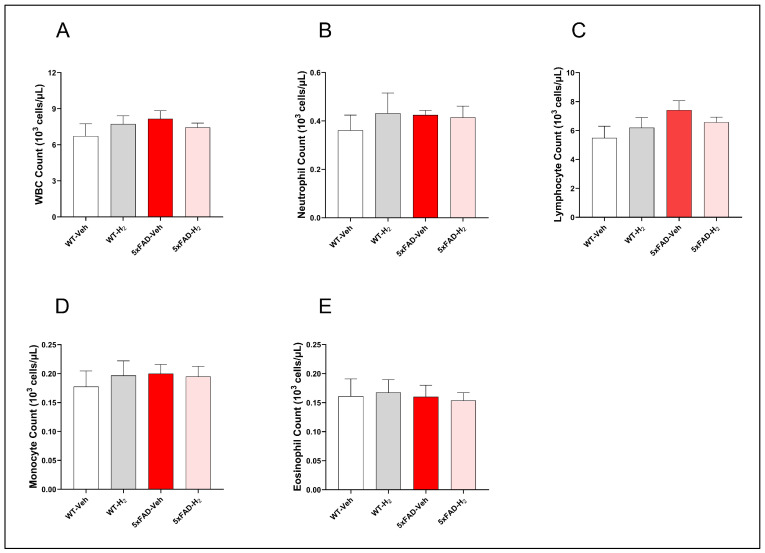
Peripheral WBC profiles in WT-Veh, WT-H_2_, 5xFAD-Veh, and 5xFAD-H_2_ mice (n = 10 per group). (**A**) Total WBC, (**B**) Neutrophil, (**C**) Lymphocyte, (**D**) Monocyte, and (**E**) Eosinophil counts in peripheral blood. Data are expressed as mean ± SD.

To determine whether the 5xFAD genotype and H_2_ inhalation altered systemic immune status, we analyzed peripheral WBC counts and differentials were across groups (Figure 2). Total WBC counts did not differ significantly among groups. However, 5xFAD-Veh mice showed a modest upward trend in total WBC counts compared with WT-Veh, WT-H_2_, and 5xFAD-H_2_ mice, primarily attributable to slightly elevated lymphocyte counts, whereas neutrophil, monocyte, and eosinophil counts remained similar across groups.

### 3.2. Effects of H_2_ in OS Markers and Antioxidant Enzyme Activities

**Figure 3:**
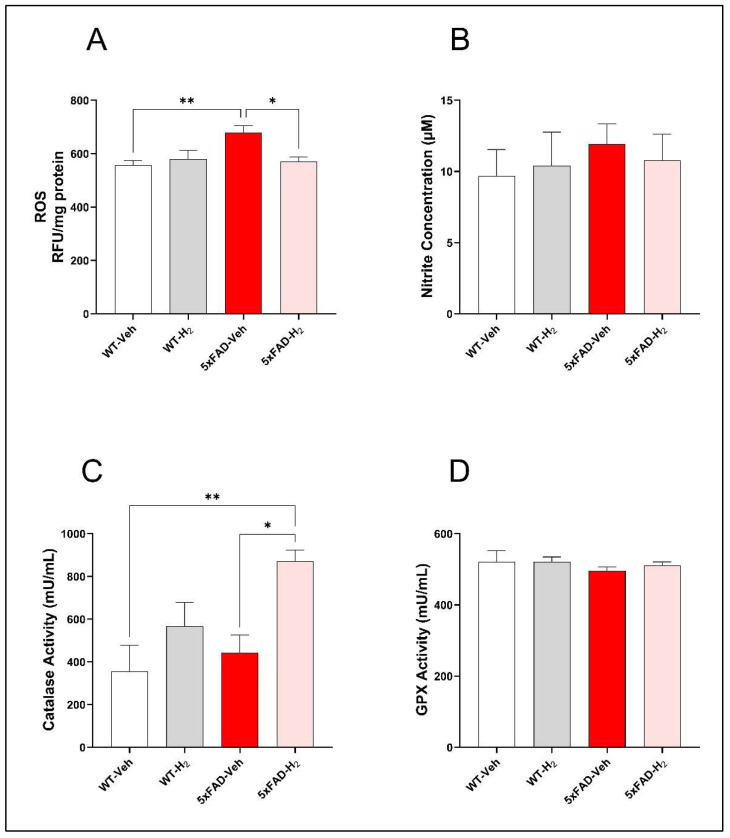
OS markers and antioxidant enzyme activities in WT-Veh, WT-H_2_, 5xFAD-Veh, and 5xFAD-H_2_ mice (n = 10 per group). (**A**) Hippocampal ROS levels. (**B**) Serum NO concentrations. (**C**) Serum catalase activity. (**D**) Serum GPX activity. Data are expressed as mean ± SD, * *p* < 0.05, ** *p* < 0.01.

To evaluate redox alterations in 5xFAD mice and their modulation by H_2_, hippocampal ROS levels and serum oxidative stress-related parameters were measured. Hippocampal ROS levels were significantly elevated in 5xFAD-Veh mice relative to WT-Veh (*p* < 0.01), and this increase was significantly attenuated by H_2_ inhalation in 5xFAD-H_2_ mice (*p* < 0.05; Figure 3A). Serum NO concentrations showed a mild upward trend in 5xFAD-Veh mice but did not differ significantly among groups (Figure 3B). In contrast, serum catalase activity was significantly increased in 5xFAD H_2_ mice compared with WT-Veh (*p* < 0.01) and 5xFAD-Veh (*p* < 0.05), indicating H_2_-induced enhancement of enzymatic antioxidant capacity (Figure 3C). Serum GPX activity did not differ among groups (Figure 3D).

### 3.3. Effects of H_2_ in Serum Cytokine Profiles

**Figure 4:**
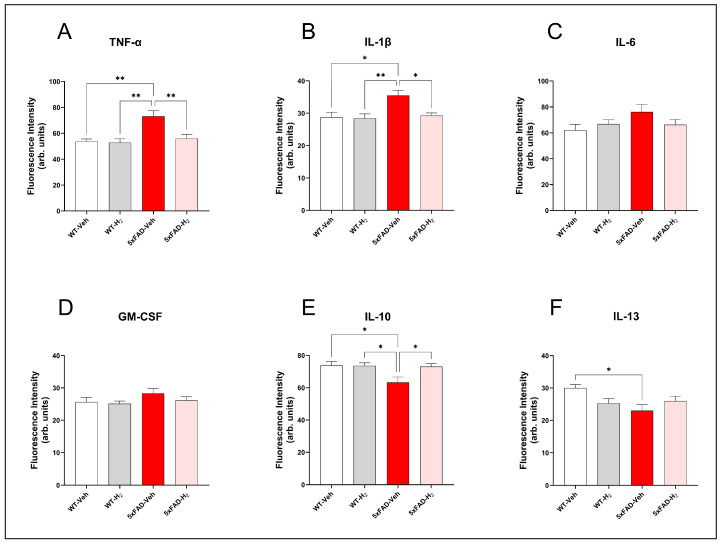
Serum cytokine profiles in WT-Veh, WT-H_2_, 5xFAD-Veh, and 5xFAD-H_2_ mice (n = 10 per group). (**A**) TNF-α. (**B**) IL-1β. (**C**) IL-6. (**D**) GM-CSF. (**E**) IL-10. (**F**) IL-13. Data are presented as mean ± SD, * *p* < 0.05, ** *p* < 0.01.

To assess systemic inflammatory changes and the effects of H_2_ treatment, serum concentrations of TNF-α, IL-1β, IL-6, GM-CSF, IL-10, and IL-13 were analyzed (Figure 4). Pro-inflammatory TNF-α and IL-1β levels were significantly elevated in 5xFAD-Veh mice compared with WT-Veh (both *p* < 0.01). H_2_ inhalation in 5xFAD-H_2_ mice normalized these cytokines to WT-Veh levels and significantly reduced them relative to 5xFAD-Veh mice (TNF-α, *p* < 0.01; IL-1β, *p* < 0.05; Figure 4A,B). IL-6 and GM-CSF levels did not differ significantly among groups, although both showed modest increases in 5xFAD-Veh mice (Figure 4C,D). In contrast, the anti-inflammatory cytokine IL-10 was significantly reduced in 5xFAD-Veh mice relative to WT-Veh (*p* < 0.05) and was restored by H_2_ inhalation in 5xFAD-H_2_ mice (*p* < 0.05; Figure 4E). IL-13 levels were also decreased in 5xFAD-Veh mice (*p* < 0.05 versus WT-Veh), and H_2_ treatment partially attenuated this reduction in 5xFAD-H_2_ mice without reaching statistical significance (Figure 4F). Collectively, these data indicate that H_2_ inhalation shifts the peripheral cytokine profile in 5xFAD mice toward a less pro-inflammatory, IL-10 dominant state.

### 3.4. Effects of H_2_ in Hippocampal Protein Expression by Western Blot

**Figure 5:**
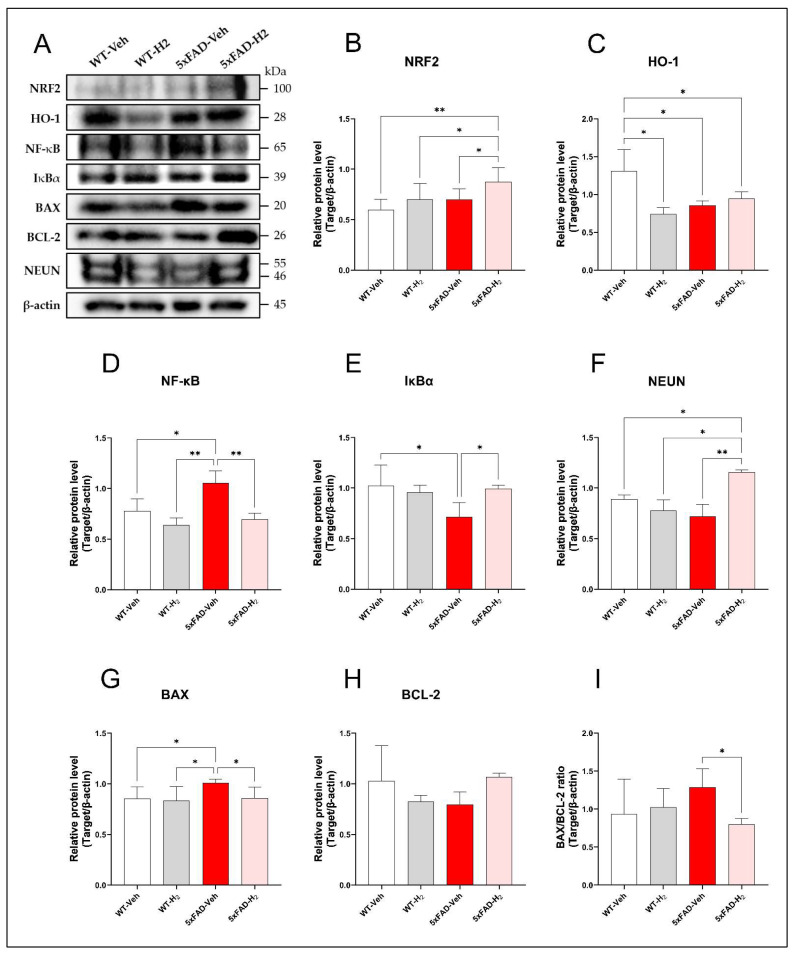
Hippocampal protein expression profiles in WT-Veh, WT-H_2_, 5xFAD-Veh, and 5xFAD-H_2_ mice (n = 10 per group). (**A**) Representative Western blots for NRF2, HO-1, NF-κB, IκBα, BAX, BCL-2, NEUN, and β-actin. (**B**) Quantification of NRF2. (**C**) Quantification of HO-1. (**D**) Quantification of NF-κB. (**E**) Quantification of IκBα. (**F**) Quantification of NEUN. (**G**) Quantification of BAX. (**H**) Quantification of BCL-2. (**I**) BAX/BCL-2 ratio. Data are presented as mean ± SD, * *p* < 0.05, ** *p* < 0.01.

To evaluate the effects of H_2_ on hippocampal oxidative stress, inflammatory signaling, and neuronal integrity in 5xFAD mice, protein expression of NRF2, HO-1, NF-κB, IκBα, BAX, BCL-2, and NEUN was analyzed by Western blotting (Figure 5). Relative to WT-Veh mice, 5xFAD-Veh mice showed significantly reduced hippocampal HO-1 expression (*p* < 0.05). H_2_ inhalation significantly increased NRF2 levels in 5xFAD-H_2_ mice compared with the 5xFAD-Veh mice (*p* < 0.05), while HO-1 expression showed a parallel upward trend, consistent with activation of the NRF2/HO-1 antioxidant pathway (Figure 5A–C). Regarding inflammatory signaling, NF-κB expression was significantly elevated in 5xFAD-Veh mice compared with WT-Veh (*p* < 0.05) and 5xFAD-H_2_ mice (*p* < 0.01) accompanied by a concomitant reduction in IκBα (*p* < 0.05). H_2_ inhalation attenuated NF-κB upregulation and partially restored IκBα levels in 5xFAD-H_2_ mice (Figure 5A,D,E), indicating suppression of hippocampal NF-κB signaling. Markers of apoptosis and neuronal integrity further supported H_2_-mediated neuroprotection. Compared with WT-H_2_ mice, 5xFAD-Veh mice exhibited increased BAX expression (*p* < 0.05) and reduced BCL-2 expression, whereas H_2_ treatment in 5xFAD-H_2_ mice decreased BAX, increased BCL-2, and significantly reduced the BAX/BCL-2 ratio (*p* < 0.05; Figure 5A,G–I). NEUN expression showed a declining trend in 5xFAD-Veh mice, while H_2_ inhalation significantly increased NEUN levels in 5xFAD-H_2_ mice (*p* < 0.01), consistent with preservation of hippocampal neuronal integrity (Figure 5F).

### 3.5. Effects of H_2_ in Hippocampal Aβ40 and Aβ42 Levels

**Figure 6:**
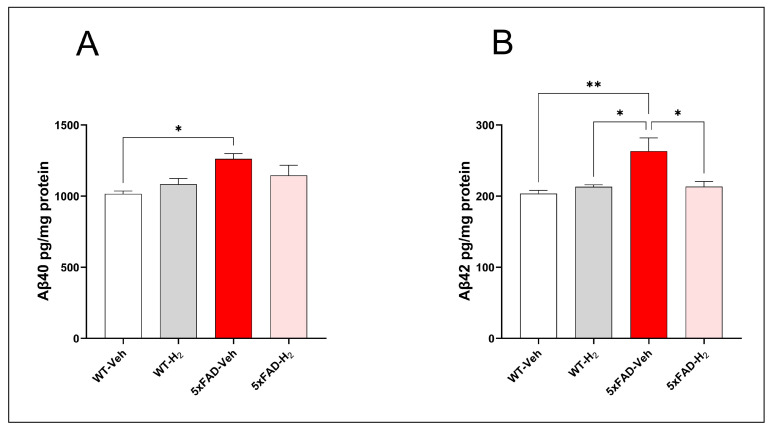
Hippocampal Aβ levels in WT-Veh, WT-H_2_, 5xFAD-Veh, and 5xFAD-H_2_ mice (n = 10 per group). (**A**) Hippocampal Aβ40 concentrations. (**B**) Hippocampal Aβ42 concentrations. Data are presented as mean ± SD, * *p* < 0.05, ** *p* < 0.01.

Hippocampal Aβ40 and Aβ42 concentrations were quantified as indices of regional amyloid burden. Consistent with the aggressive amyloidogenic phenotype of the 5xFAD model, 5xFAD-Veh mice exhibited significantly higher Aβ40 levels than WT-Veh mice (*p* < 0.05). H_2_ inhalation in 5xFAD-H_2_ mice reduced Aβ40 levels relative to 5xFAD-Veh mice, although this decrease did not reach statistical significance (Figure 6A). Aβ42 levels showed a similar but more pronounced pattern, with marked elevation in 5xFAD-Veh mice compared with WT-Veh (*p* < 0.01). In contrast, H_2_ treatment in 5xFAD-H_2_ mice normalized Aβ42 levels and significantly reduced them relative to 5xFAD-Veh mice (*p* < 0.05), indicating that H_2_ inhalation limits hippocampal accumulation of the more aggregation-prone Aβ42 species (Figure 6B).

### 3.6. Effects of H_2_ in Hippocampal ATP Levels

**Figure 7:**
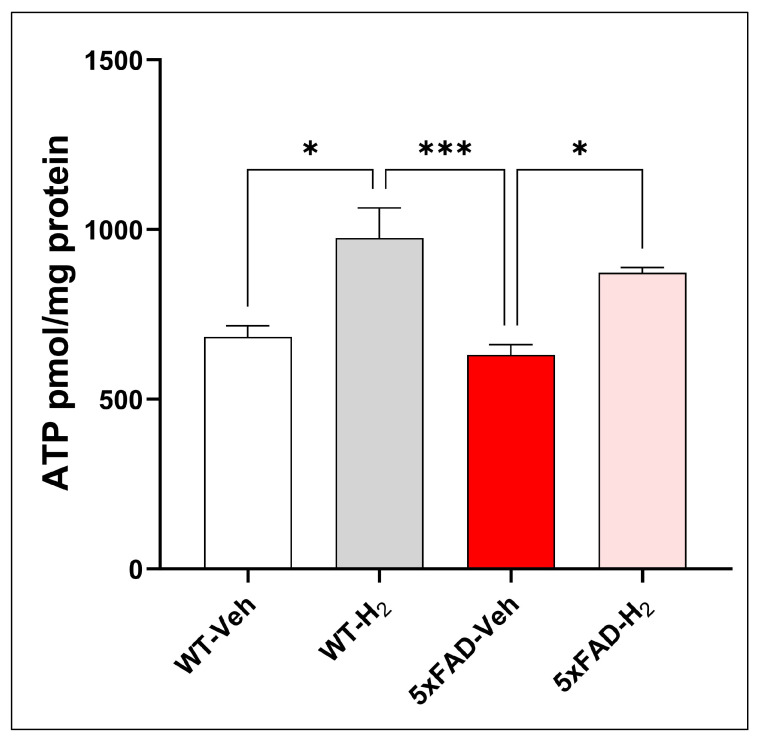
Hippocampal ATP levels in WT-Veh, WT-H_2_, 5xFAD-Veh, and 5xFAD-H_2_ mice (n = 10 per group). Data are presented as mean ± SD, * *p* < 0.05, *** *p* < 0.001.

Hippocampal ATP levels were quantified as an index of mitochondrial bioenergetic capacity. ATP levels differed significantly among groups, indicating genotype- and treatment-dependent effects on mitochondrial energy metabolism (Figure 7). Compared with WT-Veh mice, WT-H_2_ mice showed significantly increased hippocampal ATP levels (*p* < 0.05), suggesting enhanced mitochondrial bioenergetic capacity under physiological conditions. In contrast, 5xFADV-Veh mice exhibited reduced ATP levels relative to WT-H_2_ mice, consistent with impaired mitochondrial function in amyloid-bearing animals. Notably, H_2_ administration in 5xFAD-H_2_ mice significantly increased ATP levels compared with 5xFAD-Veh mice (*p* < 0.05) and partially restored ATP toward levels observed in WT-H_2_ mice, indicating mitigation of amyloid-associated mitochondrial dysfunction and partial normalization of cellular energy status.

## 4. Discussion

In the present study, our findings demonstrate that H_2_ inhalation confers broad neuroprotective effects in 5xFAD mice through modulation of redox homeostasis, inflammatory signaling, mitochondrial bioenergetics, and amyloid pathology. H_2_ treatment significantly reduced hippocampal oxidative stress and Aβ42 accumulation while preserving mitochondrial ATP production and neuronal integrity. The reduction in hippocampal Aβ42 burden, together with improved bioenergetic capacity and neuronal preservation, indicates that H_2_ alleviates upstream contributors to amyloidogenesis and mitochondrial dysfunction, yielding structural and functional neuroprotection. Moreover, the concurrent improvement in redox balance and inflammatory profiles suggests that H_2_ may disrupt pathogenic neuroimmune-metabolic interactions that promote amyloid accumulation and synaptic dysfunction. Collectively, these findings support H_2_ as a multi-target therapeutic approach for attenuating amyloid-driven neurodegeneration.

In the 5xFAD transgenic mice used in this study, early and robust amyloid deposition is accompanied by mitochondrial dysfunction and OS, which together contribute to synaptic impairment and neuronal loss [48,49]. OS is a key driver of neurodegeneration in AD. The Nrf2/HO-1 pathway acts as a central regulator of cellular redox homeostasis, and dysfunction of this axis has been implicated in AD pathogenesis. Previous studies have shown that H_2_ selectively neutralizes highly reactive oxidants such as hydroxyl radicals and ONOO^−^, thereby protecting cells from oxidative damage [30,47,50]. Excessive ROS and reactive nitrogen species can promote membrane damage, structural alterations, and ultimately cell death [49]. One of the previous studies have shown hippocampal ROS levels were significantly elevated in 5xFAD-Veh mice [51], indicating pronounced redox imbalance in this model. Our results showed H_2_ inhalation attenuated OS and was associated with increased catalase activity, while GPX activity remained unchanged. Although GPX induction after H_2_ treatment has been reported in other models [42,51], the lack of additional GPX activation here may indicate that catalase provides a rapid first-line antioxidant response once ROS levels decline [52,53]. In parallel, our results showed H_2_ inhalation increased NRF2 expression and was accompanied by a trend toward higher HO-1 expression. Because HO-1 and catalase are NRF2/ARE-regulated enzymes involved in heme metabolism and peroxide detoxification [54,55], the coordinated increase in NRF2 signaling, HO-1 expression, and catalase activity supports activation of an NRF2-mediated antioxidant response. In addition, serum NO levels in our study showed a modest upward trend in 5xFAD-Veh mice, whereas H_2_ inhalation was associated with a slight reduction, suggesting that nitrosative stress in this model is likely driven primarily by localized NO activity and protein nitration rather than systemic NO elevation. Collectively, these findings indicate that H_2_ exerts protective effects in 5xFAD mice by mitigating oxidative stress through activation of the NRF2/ARE antioxidant pathway, while also modulating inflammatory signaling.

OS and inflammation are closely interconnected process in AD pathogenesis, and redox imbalance can further amplify inflammatory signaling pathways. Parallel to these redox-directed effects, the present findings demonstrate a robust impact of H_2_ on inflammatory signaling. The 5xFAD model is characterized by chronic neuroinflammation driven by mitochondrial damage, microglial activation, and engagement of innate immune pathways, which together constitute a core component of AD pathology [6]. In this study, 5xFAD-Veh mice exhibited a peripheral cytokine profile dominated by elevated pro-inflammatory mediators TNF-α and IL-1β, accompanied by reduced levels of the anti-inflammatory cytokines IL-10 and IL-13, indicating a shift toward a strongly pro-inflammatory systemic state [56]. H_2_ inhalation largely reversed this imbalance by reducing TNF-α and IL-1β to levels comparable with WT-Veh mice, restoring IL-10, and partially normalizing IL-13, consistent with re-establishment of peripheral immune homeostasis and reinforcement of anti-inflammatory signaling. However, because inflammatory markers were quantified only in serum and not in hippocampal tissue, these results primarily reflect systemic immune modulation. Future studies incorporating hippocampal cytokine profiling would better determine whether peripheral changes correspond to local neuroinflammatory regulation within the brain. In parallel with these systemic effects, our results indicated increased hippocampal NF-κB expression and decreased IκBα levels in 5xFAD-Veh mice, whereas H_2_ treatment attenuated NF-κB activation and partially restored IκBα expression. These findings are consistent with previous reports that H_2_ suppresses NF-κB-dependent inflammatory signaling and modulates microglial activation [57,58]. Together, these observations suggest that H_2_ exerts coordinated peripheral and central anti-inflammatory effects in 5xFAD mice, likely through attenuation of mitochondrial ROS-driven immune activation and promotion of a more regulatory immune environment [59].

Importantly, these redox and inflammatory changes were accompanied by improvements in amyloid burden and neuronal integrity. The 5xFAD model is characterized by early and robust Aβ deposition [48], which was reflected in our study by markedly elevated hippocampal Aβ40 and Aβ42 levels in 5xFAD-Veh mice. In this context, H_2_ inhalation significantly reduced hippocampal Aβ42 levels and showed a modest reduction in Aβ40, without substantially altering the Aβ42/Aβ40 ratio. This selective reduction in Aβ42 is notable because Aβ42 exhibits a higher aggregation tendency and greater neurotoxicity than Aβ40, and is considered a principal driver of plaque formation and synaptic dysfunction in AD [48]. Accumulating evidence indicates that OS and neuroinflammation facilitate Aβ production and aggregation by influencing APP processing, γ-secretase activity, and clearance pathways [60,61]. By attenuating ROS accumulation and NF-κB signaling, H_2_ may indirectly limit amyloidogenic processing and enhance Aβ clearance, thereby reducing hippocampal Aβ42, which is more aggregation-prone and neurotoxic than Aβ40. In addition, normalization of TNF-α and IL-1β together with restoration of IL-10 may improve microglial phagocytic capacity and promote Aβ clearance, consistent with reports linking inflammatory phenotypes to changes in Aβ plaque burden and morphology [62].

Beyond effects of H_2_ on amyloid pathology and inflammatory signaling, mitochondrial dysfunction represents another key pathological feature of AD that contributes to neuronal injury and synaptic failure. H_2_ inhalation significantly increased hippocampal ATP levels in both wild-type and 5xFAD mice, addressing mitochondrial bioenergetic failure—an early, persistent AD feature that contributes to synaptic dysfunction and reduced neuronal resilience [63]. The observed enhancement of ATP production indicates that H_2_ not only limits oxidative injury but also improves mitochondrial function under both physiological and amyloid-associated conditions. Previous mechanistic studies have demonstrated that H_2_ stabilizes mitochondrial membrane potential, modulates electron transport chain activity, and suppresses excessive superoxide generation at complex I [32,36]. Extending these observations to an amyloid-driven context, the present findings suggest that H_2_ supports restoration of hippocampal energy metabolism and thereby promotes neuronal viability. Consistent with this interpretation, our results indicate H_2_ treatment significantly reduced the BAX/BCL-2 ratio and restored NEUN expression in 5xFAD mice, representing attenuation of pro-apoptotic signaling and preservation of neuronal populations [64,65]. Taken together with the NRF2/ARE activation and restoration of ATP levels, these findings suggest that H_2_ may function as a regulator of mitochondria-dependent apoptotic vulnerability in amyloid-driven AD pathology.

Although the present study provides mechanistic insight into H_2_-mediated neuroprotection, some methodological and experimental limitations should be considered when interpreting the findings. First, the analyses focused on biochemical and molecular outcomes without direct assessment of learning and cognitive function, which is essential for establishing functional relevance in AD models. Incorporation of behavioral paradigms, including the Morris water maze, Y-maze, and novel object recognition would strengthen translational interpretation of H_2_ efficacy. Second, due to the limited amount of hippocampal protein available, Western blot analyses were performed using combined lysates within each group samples. While this approach helped us to reduce lane-to-lane variability and limit animal use, it does not reflect true inter-individual variability or allow analysis of associations with behavioral or pathological outcomes. Therefore, this limitation should be addressed in future studies by analyzing protein expression in individual samples. Third, the study examined a single H_2_ concentration and treatment duration (2% H_2_ for 1 h daily over 4 weeks). Future studies incorporating dose–response and time-course analyses will be necessary to determine optimal therapeutic parameters. Further investigation is also needed to explain the molecular pathways responsive to H_2_ and to better define its pharmacological potential in AD-related neuroprotection.

## 5. Conclusions

In summary, this study demonstrates that H_2_ inhalation in 5xFAD mice attenuates oxidative stress, suppresses systemic and hippocampal inflammatory responses, enhances mitochondrial ATP production, reduces hippocampal Aβ42 burden, and preserves neuronal integrity. These effects are accompanied by activation of the NRF2/ARE antioxidant pathway and attenuation of NF-κB-mediated inflammatory signaling, indicating a coordinated shift toward a more permissive, neuroprotective environment. Given its favorable safety profile and capacity to target multiple converging pathways implicated in AD pathogenesis, H_2_ represents a promising adjunctive strategy for disease modification and prevention. Building on these findings, future studies incorporating behavioral outcomes, longitudinal imaging, and pathway-directed interventions will be critical to validate these protective effects and to define how H_2_ may be integrated into broader therapeutic frameworks for AD.

## Data Availability

The original contributions presented in this study are included in the article. Further inquiries can be directed to the corresponding authors.

## Abbreviations

AD	Alzheimer’s disease
ANOVA	Analysis of variance
APP	Amyloid precursor protein
ARE	Antioxidant response element
Aβ	Amyloid-beta
BAX	BCL-2-associated X protein
BCA	Bicinchoninic acid
BCL-2	B-cell lymphoma 2
cGAS	Cyclic GMP–AMP synthase
DAMPs	Damage-associated molecular patterns
DCF-DA	2′,7′-Dichlorodihydrofluorescein diacetate
EDTA	Ethylenediaminetetraacetic acid
ELISA	Enzyme-linked immunosorbent assay
GM-CSF	Granulocyte-macrophage colony-stimulating factor
GPX	Glutathione peroxidase
H_2_	Molecular hydrogen
HO-1	Heme oxygenase-1
HRP	Horseradish peroxidase
IκBα	Inhibitor of nuclear factor kappa-B alpha
IL-1β	Interleukin-1β
IL-6	Interleukin-6
IL-10	Interleukin-10
IL-13	Interleukin-13
NEUN	Neuronal nuclei
NF-κB	Nuclear factor kappa B
NLRP3	NLR family pyrin domain containing 3
NO	Nitric oxide
NRF2	Nuclear factor erythroid 2-related factor 2
OS	Oxidative stress
PBS	Phosphate-buffered saline
PMSF	Phenylmethylsulfonyl fluoride
PSEN1	Presenilin 1
PVDF	Polyvinylidene fluoride
RIPA	Radioimmunoprecipitation assay
ROS	Reactive oxygen species
SD	Standard deviation
SDS-PAGE	Sodium dodecyl sulfate polyacrylamide gel electrophoresis
TLRs	Toll-like receptors
TNF-α	Tumor necrosis factor-α
WBCs	White blood cells

## References

[1] Malaiya A., Singhai M., Singh M., Prajapati S.K., Choudhury H., Fatima M., Alexander A., Dubey S.K., Greish K., Kesharwani P. (2022). Recent update on the Alzheimer’s disease progression, diagnosis and treatment approaches. Curr. Drug Targets.

[2] Better M.A. (2023). Alzheimer’s disease facts and figures. Alzheimers Dement..

[3] Prince M., Wimo A., Guerchet M., Ali G.-C., Wu Y.-T., Prina M. (2015). World Alzheimer report 2015. The Global Impact of Dementia: An Analysis of Prevalence, Incidence, Cost and Trends.

[4] Sengoku R. (2020). Aging and Alzheimer’s disease pathology. Neuropathology.

[5] Polanco J.C., Li C., Bodea L.-G., Martinez-Marmol R., Meunier F.A., Götz J. (2018). Amyloid-β and tau complexity—Towards improved biomarkers and targeted therapies. Nat. Rev. Neurol..

[6] Jurcău M.C., Andronie-Cioara F.L., Jurcău A., Marcu F., Ţiț D.M., Pașcalău N., Nistor-Cseppentö D.C. (2022). The link between oxidative stress, mitochondrial dysfunction and neuroinflammation in the pathophysiology of Alzheimer’s disease: Therapeutic implications and future perspectives. Antioxidants.

[7] Picca A., Calvani R., Coelho-Junior H.J., Landi F., Bernabei R., Marzetti E. (2020). Mitochondrial dysfunction, oxidative stress, and neuroinflammation: Intertwined roads to neurodegeneration. Antioxidants.

[8] Perluigi M., Di Domenico F., Butterfield D.A. (2024). Oxidative damage in neurodegeneration: Roles in the pathogenesis and progression of Alzheimer disease. Physiol. Rev..

[9] Misrani A., Tabassum S., Yang L. (2021). Mitochondrial Dysfunction and Oxidative Stress in Alzheimer’s Disease. Front. Aging Neurosci..

[10] Huang Z., Chen Y., Zhang Y. (2020). Mitochondrial reactive oxygen species cause major oxidative mitochondrial DNA damages and repair pathways. J. Biosci..

[11] Su L.-J., Zhang J.-H., Gomez H., Murugan R., Hong X., Xu D., Jiang F., Peng Z.-Y. (2019). Reactive oxygen species-induced lipid peroxidation in apoptosis, autophagy, and ferroptosis. Oxidative Med. Cell. Longev..

[12] Rottenberg H., Hoek J.B. (2017). The path from mitochondrial ROS to aging runs through the mitochondrial permeability transition pore. Aging Cell.

[13] Ngo V., Duennwald M.L. (2022). Nrf2 and Oxidative Stress: A General Overview of Mechanisms and Implications in Human Disease. Antioxidants.

[14] Ali J., Choe K., Park J.S., Park H.Y., Kang H., Park T.J., Kim M.O. (2024). The Interplay of Protein Aggregation, Genetics, and Oxidative Stress in Alzheimer’s Disease: Role for Natural Antioxidants and Immunotherapeutics. Antioxidants.

[15] Koenig A., Buskiewicz-Koenig I.A. (2022). Redox Activation of Mitochondrial DAMPs and the Metabolic Consequences for Development of Autoimmunity. Antioxid. Redox Signal..

[16] Mun Y., Kim J., Choi Y.-J., Lee B.-H. (2025). cGAS–STING–NF-κB Axis Mediates Rotenone-Induced NLRP3 Inflammasome Activation Through Mitochondrial DNA Release. Antioxidants.

[17] Bauernfeind F.G., Horvath G., Stutz A., Alnemri E.S., MacDonald K., Speert D., Fernandes-Alnemri T., Wu J., Monks B.G., Fitzgerald K.A. (2009). Cutting edge: NF-κB activating pattern recognition and cytokine receptors license NLRP3 inflammasome activation by regulating NLRP3 expression. J. Immunol..

[18] Franchi L., Eigenbrod T., Núñez G. (2009). Cutting edge: TNF-α mediates sensitization to ATP and silica via the NLRP3 inflammasome in the absence of microbial stimulation. J. Immunol..

[19] Graham W.V., Bonito-Oliva A., Sakmar T.P. (2017). Update on Alzheimer’s Disease Therapy and Prevention Strategies. Annu. Rev. Med..

[20] Cummings J.L. (2025). Maximizing the benefit and managing the risk of anti-amyloid monoclonal antibody therapy for Alzheimer’s disease: Strategies and research directions. Neurotherapeutics.

[21] Congdon E.E., Ji C., Tetlow A.M., Jiang Y., Sigurdsson E.M. (2023). Tau-targeting therapies for Alzheimer disease: Current status and future directions. Nat. Rev. Neurol..

[22] Hossain M.S., Hussain M.H. (2025). Multi-Target Drug Design in Alzheimer’s Disease Treatment: Emerging Technologies, Advantages, Challenges, and Limitations. Pharmacol. Res. Perspect..

[23] Lau S.-F., Wu W., Wong H.Y., Ouyang L., Qiao Y., Xu J., Lau J.H.-Y., Wong C., Jiang Y., Holtzman D.M. (2023). The VCAM1–ApoE pathway directs microglial chemotaxis and alleviates Alzheimer’s disease pathology. Nat. Aging.

[24] Nguyen A.T., Wang K., Hu G., Wang X., Miao Z., Azevedo J.A., Suh E., Van Deerlin V.M., Choi D., Roeder K. (2023). Correction: APOE and TREM2 regulate amyloid-responsive microglia in Alzheimer’s disease. Acta Neuropathol..

[25] Gao C., Jiang J., Tan Y., Chen S. (2023). Microglia in neurodegenerative diseases: Mechanism and potential therapeutic targets. Signal Transduct. Target. Ther..

[26] Xu K., Sun G., Wang Y., Luo H., Wang Y., Liu M., Liu H., Lu X., Qin X. (2024). Mitigating radiation-induced brain injury via NLRP3/NLRC4/Caspase-1 pyroptosis pathway: Efficacy of memantine and hydrogen-rich water. Biomed. Pharmacother..

[27] Iketani M., Ohsawa I. (2017). Molecular hydrogen as a neuroprotective agent. Curr. Neuropharmacol..

[28] Wu C., Zou P., Feng S., Zhu L., Li F., Liu T.C., Duan R., Yang L. (2023). Molecular Hydrogen: An Emerging Therapeutic Medical Gas for Brain Disorders. Mol. Neurobiol..

[29] Chen W., Zhang H.T., Qin S.C. (2021). Neuroprotective Effects of Molecular Hydrogen: A Critical Review. Neurosci. Bull..

[30] Ohsawa I., Ishikawa M., Takahashi K., Watanabe M., Nishimaki K., Yamagata K., Katsura K.-i., Katayama Y., Asoh S., Ohta S. (2007). Hydrogen acts as a therapeutic antioxidant by selectively reducing cytotoxic oxygen radicals. Nat. Med..

[31] Radyuk S.N. (2021). Mechanisms underlying the biological effects of molecular hydrogen. Curr. Pharm. Des..

[32] Zhang X., Xie F., Ma S., Ma C., Jiang X., Yi Y., Song Y., Liu M., Zhao P., Ma X. (2023). Mitochondria: One of the vital hubs for molecular hydrogen’s biological functions. Front. Cell Dev. Biol..

[33] Johnsen H.M., Hiorth M., Klaveness J. (2023). Molecular Hydrogen Therapy—A Review on Clinical Studies and Outcomes. Molecules.

[34] Ishihara G., Kawamoto K., Komori N., Ishibashi T. (2020). Molecular hydrogen suppresses superoxide generation in the mitochondrial complex I and reduced mitochondrial membrane potential. Biochem. Biophys. Res. Commun..

[35] Zhang J., Zhou Y., Zhu X., Peng Z., Zhu Q., Ding P., Ning M., Zhang Q.R., Zhuang Z. (2025). Hydrogen Attenuates Oxidative Damage via NRF2-Mediated Mitophagy after Subarachnoid Hemorrhage. Antioxid. Redox Signal.

[36] Cheng D., Long J., Zhao L., Liu J. (2023). Hydrogen: A Rising Star in Gas Medicine as a Mitochondria-Targeting Nutrient via Activating Keap1-Nrf2 Antioxidant System. Antioxidants.

[37] Liu Y., Dong F., Guo R., Zhang Y., Qu X., Wu X., Yao R. (2019). Hydrogen-Rich Saline Ameliorates Experimental Autoimmune Encephalomyelitis in C57BL/6 Mice Via the Nrf2-ARE Signaling Pathway. Inflammation.

[38] Shi Y., Wang G., Li J., Yu W. (2017). Hydrogen gas attenuates sevoflurane neurotoxicity through inhibiting nuclear factor κ-light-chain-enhancer of activated B cells signaling and proinflammatory cytokine release in neonatal rats. Neuroreport.

[39] Huang J.-L., Liu W.-W., Manaenko A., Sun X.-J., Mei Q.-Y., Hu Q. (2019). Hydrogen inhibits microglial activation and regulates microglial phenotype in a mouse middle cerebral artery occlusion model. Med. Gas. Res..

[40] Abdul-Nasir S., Chau C.T., Nguyen T.T., Bajgai J., Rahman M.H., Hwang-Un K., You I.-S., Kim C.-S., Seo B.A., Lee K.-J. (2025). Hydrogen Gas Attenuates Toxic Metabolites and Oxidative Stress-Mediated Signaling to Inhibit Neurodegeneration and Enhance Memory in Alzheimer’s Disease Models. Int. J. Mol. Sci..

[41] Hirano S.I., Aoki Y., Li X.K., Ichimaru N., Takahara S., Takefuji Y. (2021). Protective effects of hydrogen gas inhalation on radiation-induced bone marrow damage in cancer patients: A retrospective observational study. Med. Gas. Res..

[42] He W., Rahman M.H., Bajgai J., Abdul-Nasir S., Mo C., Ma H., Goh S.H., Bomi K., Jung H., Kim C.-S. (2024). Hydrogen Gas Inhalation Alleviates Airway Inflammation and Oxidative Stress on Ovalbumin-Induced Asthmatic BALB/c Mouse Model. Antioxidants.

[43] Tian Y., Zhang Y., Wang Y., Chen Y., Fan W., Zhou J., Qiao J., Wei Y. (2021). Hydrogen, a Novel Therapeutic Molecule, Regulates Oxidative Stress, Inflammation, and Apoptosis. Front. Physiol..

[44] Jin J., Yue L., Du M., Geng F., Gao X., Zhou Y., Lu Q., Pan X. (2025). Molecular Hydrogen Therapy: Mechanisms, Delivery Methods, Preventive, and Therapeutic Application. MedComm.

[45] Dumbuya J.S., Zeng C., Ahmad B., Tian C., Lu J. (2026). The protective effects of molecular hydrogen in sepsis-associated encephalopathy: Current status. Eur. J. Pharmacol..

[46] Sugai K., Tamura T., Sano M., Uemura S., Fujisawa M., Katsumata Y., Endo J., Yoshizawa J., Homma K., Suzuki M. (2020). Daily inhalation of hydrogen gas has a blood pressure-lowering effect in a rat model of hypertension. Sci. Rep..

[47] Jeong E.S., Bajgai J., You I.S., Rahman M.H., Fadriquela A., Sharma S., Kwon H.U., Lee S.Y., Kim C.S., Lee K.J. (2021). Therapeutic Effects of Hydrogen Gas Inhalation on Trimethyltin-Induced Neurotoxicity and Cognitive Impairment in the C57BL/6 Mice Model. Int. J. Mol. Sci..

[48] Oakley H., Cole S.L., Logan S., Maus E., Shao P., Craft J., Guillozet-Bongaarts A., Ohno M., Disterhoft J., Van Eldik L. (2006). Intraneuronal beta-amyloid aggregates, neurodegeneration, and neuron loss in transgenic mice with five familial Alzheimer’s disease mutations: Potential factors in amyloid plaque formation. J. Neurosci..

[49] Zhong M.Z., Peng T., Duarte M.L., Wang M., Cai D. (2024). Updates on mouse models of Alzheimer’s disease. Mol. Neurodegener..

[50] Wang Z., Feng W., Li X., Yun X., Wu S., Du L., Wang H. (2026). Targeting the Nrf2/HO-1 aixs: A therapeutic strategy against regulated cell death in Alzheimer’s disease. Ageing Res. Rev..

[51] You I.S., Sharma S., Fadriquela A., Bajgai J., Thi T.T., Rahman M.H., Sung J., Kwon H.U., Lee S.Y., Kim C.S. (2021). Antioxidant Properties of Hydrogen Gas Attenuates Oxidative Stress in Airway Epithelial Cells. Molecules.

[52] Milgrom L.R. (2016). Why is catalase so fast? A preliminary network hypothesis for the rapid enzyme-catalysed decomposition of hydrogen peroxide. Water.

[53] Nandi A., Yan L.J., Jana C.K., Das N. (2019). Role of Catalase in Oxidative Stress- and Age-Associated Degenerative Diseases. Oxidative Med. Cell. Longev..

[54] Loboda A., Damulewicz M., Pyza E., Jozkowicz A., Dulak J. (2016). Role of Nrf2/HO-1 system in development, oxidative stress response and diseases: An evolutionarily conserved mechanism. Cell. Mol. Life Sci..

[55] Jenkins T., Gouge J. (2021). Nrf2 in cancer, detoxifying enzymes and cell death programs. Antioxidants.

[56] Al-Qahtani A.A., Alhamlan F.S., Al-Qahtani A.A. (2024). Pro-inflammatory and anti-inflammatory interleukins in infectious diseases: A comprehensive review. Trop. Med. Infect. Dis..

[57] Chen H., Dong B., Shi Y., Yu Y., Xie K. (2021). Hydrogen Alleviates Neuronal Injury and Neuroinflammation Induced by Microglial Activation via the Nuclear Factor Erythroid 2-related Factor 2 Pathway in Sepsis-associated Encephalopathy. Neuroscience.

[58] Hu Y., Wang P., Han K. (2022). Hydrogen Attenuated Inflammation Response and Oxidative in Hypoxic Ischemic Encephalopathy via Nrf2 Mediated the Inhibition of NLRP3 and NF-κB. Neuroscience.

[59] Bhol N.K., Bhanjadeo M.M., Singh A.K., Dash U.C., Ojha R.R., Majhi S., Duttaroy A.K., Jena A.B. (2024). The interplay between cytokines, inflammation, and antioxidants: Mechanistic insights and therapeutic potentials of various antioxidants and anti-cytokine compounds. Biomed. Pharmacother..

[60] Cheignon C., Tomas M., Bonnefont-Rousselot D., Faller P., Hureau C., Collin F. (2018). Oxidative stress and the amyloid beta peptide in Alzheimer’s disease. Redox Biol..

[61] Zhang Y., Chen H., Li R., Sterling K., Song W. (2023). Amyloid β-based therapy for Alzheimer’s disease: Challenges, successes and future. Signal Transduct. Target. Ther..

[62] Ghimire A., Rehman S.A., Subhani A., Khan M.A., Rahman Z., Iqubal M.K., Iqubal A. (2025). Mechanism of microglia-mediated neuroinflammation, associated cognitive dysfunction, and therapeutic updates in Alzheimer’s disease. hLife.

[63] D’Alessandro M.C.B., Kanaan S., Geller M., Praticò D., Daher J.P.L. (2025). Mitochondrial dysfunction in Alzheimer’s disease. Ageing Res. Rev..

[64] Schroer J., Warm D., De Rosa F., Luhmann H.J., Sinning A. (2023). Activity-dependent regulation of the BAX/BCL-2 pathway protects cortical neurons from apoptotic death during early development. Cell. Mol. Life Sci..

[65] Moon J., Ahn J.H., Won M.-H. (2026). NeuN expression in health and disease: A histological perspective on neuronal heterogeneity. Histol. Histopathol..

